# Implementing guidelines for depression on antidepressant prescribing in general practice: a quasi-experimental evaluation

**DOI:** 10.1186/1471-2296-15-35

**Published:** 2014-02-19

**Authors:** Gerdien Franx, Jochanan Huyser, Jan Koetsenruijter, Christina M van der Feltz-Cornelis, Peter FM Verhaak, Richard PTM Grol, Michel Wensing

**Affiliations:** 1 Trimbos Institute, Netherlands institute of mental health and addiction, PO Box 725, 3500 AS Utrecht, the Netherlands; 2 Arkin, PO Box 75848, 1070 AV Amsterdam, the Netherlands; 3 Scientific Institute for Quality of Healthcare, Radboud University Nijmegen Medical Centre, PO Box 9101, 114, 6500 HB Nijmegen, the Netherlands; 4 Tilburg University, Tranzo, Tilburg School of Social and Behavioral Sciences, PO Box 90153, 5000 LE Tilburg, the Netherlands; 5 Clinical Centre for Body, Mind and Health, GGz Breburg, PO Box 770, 5000 AT Tilburg, the Netherlands; 6 Nivel, Netherlands institute for health services research, PO Box 1568, 3500 BN Utrecht, the Netherlands; 7 Rijksuniversiteit Universiteit Groningen, University Medical Centre Groningen, dep. General Practice, PO Box 196, FA20, 9700 AD Groningen, the Netherlands

**Keywords:** General practice, Guidelines, Antidepressants, Implementation, Stepped care

## Abstract

**Background:**

Internationally, guidelines for depression recommend a stepped care approach, implying that antidepressant medication should not be offered as a first step treatment to patients with sub-threshold or mild depression. In the Netherlands, antidepressant prescribing rates in general practice as a first treatment step are considered to be high. The aim of this study was to evaluate the implementation of guideline recommendations on antidepressant prescribing.

**Methods:**

A quasi-experimental study with a non-equivalent naturalistic control group and three years follow-up was performed in the general practice setting in the Netherlands. General Practitioners (GPs) participated in a national Quality Improvement Collaborative (QIC), focusing on the implementation of a guideline based model for a stepped care approach to depression. The model consisted of self-help and psychological treatment options for patients with milder symptoms as an alternative to antidepressants in general practice. Changes in antidepressant prescription rates of GPs were documented for a three-year period and compared to those in a control group of GPs, selected from an ongoing national registration network.

**Results:**

A decrease of 23.3% (49.4%-26.1%) in antidepressant prescription rates for newly diagnosed patients with depressive symptoms was found within the intervention group, whereas no difference occurred in the reference group (50.3%-52.6%). The decrease over time was significant, compared to the usual care group (OR 0.44, 95% CI: 0.21-0.92).

**Conclusions:**

An implementation program using stepped care principles for the allocation of depression interventions resulted in reduced antidepressant prescription rates in general practice. GPs can change prescribing behaviour within the context of a QIC.

## Background

Depression is a highly prevalent condition with a range of effective treatment options, many of which can be offered in general practice. Since 2004, guidelines in several countries recommend a ‘stepped care approach’ as a framework for organising depression care, putting treatment options in a specific order and relating them to patient severity profiles [1-5].

Derived from this framework, the national evidence-based multidisciplinary guideline for depression in the Netherlands, developed by a Guideline Development Group (GDG) consisting of GPs, psychiatrists, psychologists, allied health professionals, consumers and carers, recommended that antidepressant medication should not be offered as a first step treatment to patients with sub-threshold or mild depression. Instead, brief and non-pharmaceutical interventions including watchful waiting, (guided) self-help based on cognitive behavioural therapy (CBT), physical exercise and problem solving therapy were considered appropriate choices in the beginning of a treatment episode. Antidepressant medication or psychotherapy was to be offered as a first step treatment option to patients with moderate, severe or chronic symptoms [1,2].

Depression care, according to guideline recommendations, does not seem to be current practice. Rates of guideline-concordant care reported in the literature vary depending on setting, country and criteria for appropriateness, from 22% in the United States [6] to 42% in the Netherlands [7]. Focusing on recommendations concerning antidepressant prescribing, a number of problems exist. Firstly, antidepressant prescription rates in primary care are high in the Netherlands: 76% in 2002 and 70% in 2008 [8]. Although these rates appear to have declined in recent years, they have risen strongly over the last decades, with rises of more than 30% being reported in different countries [9-14]. One explanation for this rise of volume in antidepressant prescribing is the change in the proportion of patients receiving long term treatment [15,16]. Secondly, antidepressant prescription for depression during the first contact with the patient has also risen, from 62% of the cases in 1993 to 73% in 1998 [17]. Thirdly, there is a strong variation in prescribing between GPs, which can be explained by population, GP and practice characteristics [12]. Finally, prescription of antidepressants by GPs seems unrelated to symptom severity [18]. In addition, effective and brief, low intensity alternatives are relatively unknown as yet whereas it is considered essential that they are implemented in general practice [19]. As well, considering the fact that antidepressant treatment does not comply with the preferences of a great deal of patients, many of whom give negative reports of ineffectiveness and side-effects, there seems to be a need to change prescribing behaviour in general practice in the direction of a more stepped care approach, and in accordance with the clinical guidelines [18,20-23].

In order to bring about this change and implement key recommendations of the national depression guidelines in the Netherlands, a QIC with a three year follow-up was run from December 2006 until March 2008, as part of the National Depression Initiative [24]. QICs are multifaceted implementation strategies, offered to clinical care teams to rapidly improve performance and outcomes [25-28]. Parallel to the QIC, an implementation study was performed to determine the impact of the Depression QIC on antidepressant prescribing by GPs.

## Methods

Adopting a stepped care model for the management of depression is a major change and thus makes it difficult to allocate randomly to healthcare professionals, because of the risk of low inclusion rates and withdrawal at professional level because of discontentment with the randomization procedure. Therefore we performed a quasi-experimental evaluation with a non-equivalent naturalistic control group and a three year follow-up period.

### Study population

The study included two study groups (‘QIC’ and ‘usual care’, see below) and three measurement moments in each group. The health professionals in the intervention group were GPs (who provide all primary medical care in the Netherlands) participating in the depression QIC programme, described in detail elsewhere [29]. At the start of the QIC, all thirty-nine GPs were invited to participate in the study, alongside their implementation work. In order to participate, they had to consent to comply with data collection procedures. Practices were paid a fee for the time spent on research activities. Finally, twenty GPs consented and were included in the study.

The control group included GPs from practices participating in the Netherlands Information Network of General Practice (LINH), the principle national database in the Netherlands for general practice research, holding longitudinal and nationally representative data on morbidity, prescribing, and referrals [30]. In principle, patients receiving care as usual, had access to all types of depression treatment, including the low intensity treatments, although these were relatively unknown by primary care providers [19]. LINH physicians and the QIC physicians were considered to be proper naturalistic comparison groups, since participation in both programmes required the GPs’ commitment to register practice data for research and quality improvement purposes. LINH-practices were only included in the study if the Electronic Medical Record (EMR) provided information about at least 90% of the three years study duration.

The included patients in both groups were aged 18–65, with a newly recorded diagnosis of depression as documented by the GPs in the EMR, along with an International Classification of General Practice (ICPC) diagnosis of depressive feelings (ICPC code P03) or depression (ICPC code P76) [31-33].

Ethical approval was provided by the METIGG, a national ethics committee in mental healthcare in the Netherlands.

### Intervention

A Depression QIC was executed over fifteen months. A QIC is an implementation strategy applied in many countries for various clinical problems, and generally has five essential features: (1) a focus on a specific topic with gaps between best and current practice; (2) clinical experts providing ideas and support for improvement; (3) multidisciplinary teams from multiple sites participate; (4) there is a model for improvement (setting targets, collecting data and testing changes); and (5) a collaborative process with a series of structured activities in a given time frame [28,34,35]. These structured activities, which were offered to the participants during the Depression QIC, are listed in Table 1.

**Table 1:** Structured activities of the depression QIC

	**Structured activities offered**
**•**	A network of multidisciplinary teams
**•**	An expert team, teaching the stepped care model;
**•**	SMART goal setting, a set of indicators to monitor results and an excel worksheet;
**•**	A training for local team coordinators on breakthrough method and data collection;
**•**	Four conference days for all improvement teams for exchange and learning;
**•**	One conference day for local team coordinators for intensive exchange with the expert team;
**•**	Five meetings between local team coordinators, with the expert team present;
**•**	Team visits of experts and national project coordinators;
**•**	Telephone contact between local and national coordinators;
**•**	Written feedback on improvement reports and data charts;
**•**	A virtual network environment for exchange of best-practices, a Toolkit of instruments and treatment protocols, online discussions and links to relevant sites;
**•**	A two days training problem solving treatment for professionals;
**•**	A workshop workflow Improvement.

**Figure 1:**
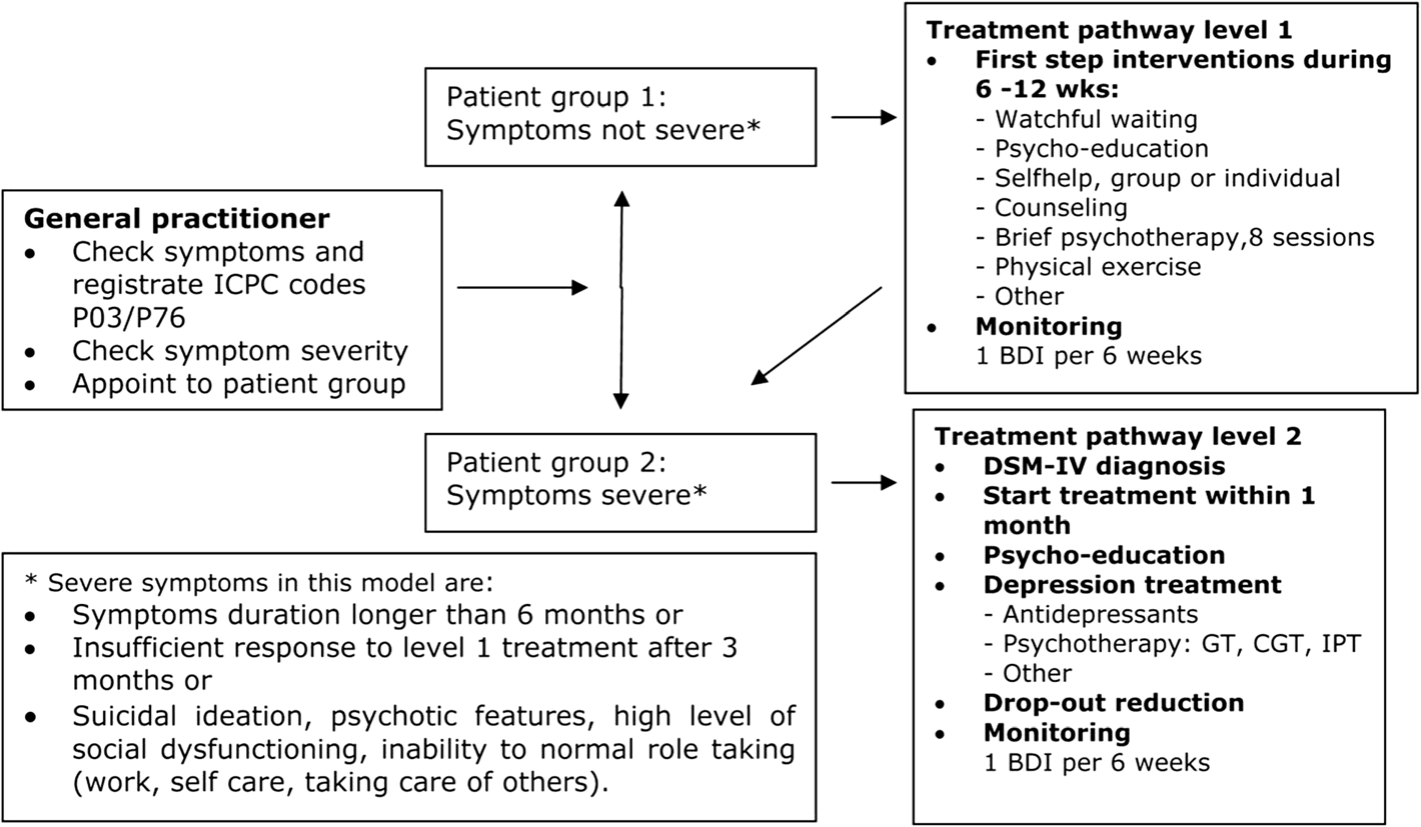
Stepped care depression model.

The focus of the Depression QIC was a stepped care model for depression treatment (see Figure 1), developed by the QIC’s clinical expert team and based on both the multidisciplinary guidelines and previous projects [1,36,37]. The model consisted of two pathways for patients with different severity profiles. Severity criteria were derived from the Diagnostic and Statistical Manual of Mental Disorders Fourth Edition (DSM IV) and based on the expert team’s opinion. Antidepressant medication was not an option in treatment pathway 1, but could be considered after a first step intervention had not resulted in sufficient treatment response. Antidepressants and psychotherapy were first line treatment options in pathway 2. The model served to guide clinicians in their improvement work.

### Data collection

The primary outcome was antidepressant prescribing, defined as the volume of antidepressant prescriptions for the depressed general practice population (prescription rates), issued by GPs as a first line treatment choice within one month after the diagnosis. The secondary outcome was referral by the GPs to clinicians providing psychological treatment. In both groups, all relevant data of patients with ICPC P03 or ICPC P76 were extracted from the EMRs of the general practices. Documentations by the physicians of co-morbid anxiety, using ICPC codes P01 (anxious, nervous, tensed feelings), P74 (anxiety disorder, condition of anxiety) and P75 (hysteria, hypochondria), were also extracted. Data extraction in the QIC group was performed by the physicians’ assistants who had received a detailed protocol for computerised searching and support from the researchers. Data extraction in the control group was performed by the staff from the LINH database.

Antidepressant medication covered the subgroup N06A of the Anatomical Therapeutic Chemical (ATC) Classification System of the World Health Organization. Referrals included a documentation of a referral to a primary care psychologist or a specialised psychologist, a psychiatrist, a psychotherapist, an institution for ambulatory care or a mental health hospital. Data collection covered a three year period: the year 2006 indicates the baseline measurement before the QIC, the year 2007 indicates the year of the intervention and the year 2008 largely indicates the follow-up measurement, after the QIC had ended.

### Data analysis

Descriptive statistics were calculated within the groups. Using a t-test, we examined the changes during the three year follow-up within both study groups. To determine the effect of participation in the implementation programme and to correct for the clustering effect, we applied a multilevel logistical regression analysis with a two-level structure with patients nested within general practices. A statistical analysis was performed in MLwiN 2.15 comparing the outcomes between the two conditions, with antidepressant prescription (yes or no) or referral to mental healthcare (yes or no) as outcomes, and the following predictors to test the difference in changes between the two conditions: group (QIC or usual care), patient age and gender, co-morbid anxiety (yes or no ICPC P01, P74, P75), year (2006, 2007, 2008) and an interaction term with year and group.

## Results

Twenty GPs from seventeen practices participated in the intervention group, and 115 GPs from forty-one practices were selected as controls. In the intervention group, the data of 400 patients were extracted for analysis, and in the control group this number was 3956.

### Characteristics of the patient population

In the QIC group, the mean age of patients with an ICPC documentation of P03 and P76 was 39.8 years, and 41.9 years in the control group. In the QIC group, the proportion of younger persons was higher (37.5% versus 32.3%), whereas the control group consisted of a larger proportion of older persons (23.3% versus 29.2%) (Table 2).

**Table 2:** Characteristics of the patient population (in percentages)

		**QIC practices (n = 400)**	**Usual care practices (n = 3956)**
Age	18-35	37.5	32.3
	36-50	39.3	38.5
	51-65	23.3	29.2
	Mean	39.8	41.9
Male		30.0	35.1
Female		70.0	64.9
Comorbid anxiety		4.0	5.8

### Antidepressant prescription

Table 3 shows the changes in professional performance within both groups in terms of antidepressant prescribing and referrals. During the three year follow up, a decrease of 23.3% in the prescription of antidepressant medication occurred in the QIC group (from 49.4% in 2006 to 26.1% in 2008). The usual care group did not change prescription rates (from 50.3% in 2006 to 52.6% in 2008).

**Table 3:** Patients with first or new depressive symptoms receiving an antidepressant prescription or being referred to mental healthcare within one month (in percentages of the total of patients with a first or new depressive episode)

	**QIC practices**			**Usual care practices**		
	**2006**	**2007**	**2008**	**2006**	**2007**	**2008**
Antidepressant prescription	49.4	32.2*	26.1*	50.3	47.0	52.6
Referral to mental healthcare	11.5	16.4	11.2	10.1	13.0*	9.0
N	87	152	161	1261	996	1699

*sign. < 0,05 compared with baseline (2006).

### Referral rates

Overall, referral rates of GPs in the QIC practices were somewhat higher than in the usual care practices during the three years study interval. In 2006, 11.5% of the patients in QIC practices were referred within a month after diagnosis to a psychologist, a primary care psychologist, a psychiatrist, a psychotherapist, an institution for ambulatory care or a mental health hospital. In 2008, this rate remained at the same level of 11.2%. With regards to baseline referral by GPs, in the usual care practices, a non-significant decline occurred from 10.1% in 2006 to 9% in 2008 (Table 3).

### Factors associated with antidepressant medication and referral to mental healthcare

Table 4 shows the factors associated with the changes between the two study groups during our study period. The usual care clinicians did not change their prescribing behaviour in 2007 (OR 0.92) or 2008 (OR 0.87). In the QIC group, antidepressant prescribing as a first line treatment option did not change in 2007, but the frequency of prescribing decreased in 2008 in almost half of the cases, compared to the usual care group (OR 0.44). QIC GPs tended to prescribe more frequently to older patients and to those who had additional anxiety problems. Multivariate analysis, however, showed that these characteristics did not account for the effect of the intervention and that participation in the QIC over time accounted for a significant decline in prescription rates (OR 0.44), compared to the usual care group. There was no significant change of referral behaviour in either group.

**Table 4:** Factors associated with antidepressant prescribing and referral to mental healthcare

		**Antidepressant prescribing**		**Referral to mental healthcare**	
		**OR (95% CI)**		**OR (95% CI)**	
Age of patient		1,03*	(1,03 - 1,04)	0,97*	(0,96 - 0,98)
Sexe of patient (male = ref)		0,98	(0,86 - 1,12)	0,71*	(0,58 - 0,85)
Co-morbid anxiety		1,66*	(1,26 - 2,18)	0,71	(0,45 - 1,13)
Participation in collaborative		0,98	(0,57 - 1,70)	0,93	(0,36 - 2,39)
Year (CAU)	2006 (ref)				
	2007	0,92	(0,69 - 1,21)	1,12	(0,68 - 1,86)
	2008	0,87	(0,66 - 1,15)	1,28	(0,77 - 2,11)
Year* collaborative	2006 (ref)				
	2007	0,60	(0,29 - 1,24)	1,11	(0,32 - 3,81)
	2008	0,44*	(0,21 - 0,92)	0,71	(0,20 - 2,52)
ICC	0.067			0.193	

*p < 0,05, n = 4356.

## Discussion and conclusions

### Summary

We found a substantial change in the professional performance of GPs participating in a quality improvement programme, in terms of lowered antidepressant prescription rates as a first step treatment choice for patients with depression. GPs providing usual care did not change their prescribing behaviour during the three year course of the study. In both groups, there was no change in referral rates to mental health clinicians.

The results seem to support the hypothesis that a QIC aimed at adherence to depression guidelines reduces antidepressant prescription rates of GPs, whereas GPs who have access to guidelines but who do not receive an intervention aimed at their implementation, don’t change their prescribing behaviour. Two other qualitative studies, performed parallel to this controlled study, showed that instead of medication, the GPs started to offer low-intensity interventions to their patients during the QIC, such as guided self-help or brief psychotherapy, and that because of these alternative treatment options they felt more at ease in reducing antidepressant prescriptions for patients with mild symptoms [29,38]. The second result of the study, the lack of a significant change in referral rates in both groups, could point at the fact that the QIC participants did not replace the medication by a more specialised psychological intervention by a primary care psychologist or a specialist in the mental health sector, but by an intervention in general practice or by ‘watchful waiting’, namely psycho-education and pro-active follow up.

### Strengths and limitations

A particular strength of this study was the evaluation of an ambitious quality improvement initiative with a direct comparison between two naturalistic groups, which makes the study appropriate to be included in an evidence review of quality improvement interventions [39]. Other strengths included the large numbers of patients and the substantial effect on the primary outcome.

A first study limitation was the lack of a randomisation procedure, which was not an option since the researchers had no control over the allocation of GPs to a particular condition. By conducting a randomised clinical trial, effects in terms of causality would have been stronger supported. However, RCTs may have the disadvantage of low inclusion rates and withdrawal at professional level because of discontentment with the randomization procedure, especially in implementation studies, thus introducing other problems of selection bias and lack of generalisibility of the results. Therefore, we considered the quasi-experimental design of this study valid for the exploration of our research question and dealt with this risk of selection bias by choosing the best possible comparison group in the Netherlands. This national database of GP performance, considered as the ‘golden standard’ for measuring care as usual because of the adequate documentation by these doctors who sign an agreement to document ICPC diagnosis and treatments provided. Unfortunately, this did not enable us to use patient reported depression outcomes, since these were not documented in the databases of routinely collected clinical data.

Another well known challenge in observational studies is the risk of bias due to confounding, which in our study could have occurred in terms of factors other than the QIC causing the observed changes. We were able to control for age, gender and co-morbid conditions of both study groups, but other factors may have played a role as well. Nevertheless, it is improbable that any one of these other factors would have caused a decline of 23% in prescription in the intervention group, which is considered to be quite substantial in the implementation literature. A second limitation of our study was the EMR data-extraction which was performed by administrative assistants in the different practices, who were very well known with the systems. We limited this risk of bias by providing all persons with the same instructions and by performing all data analysis by one research group.

### Comparison with existing literature

Our study can be compared to the Depression QIC, organised by the Institute for Healthcare Improvement in the United States in 2000–2001 and based on Wagner’s Chronic Care Model (CCM) [40]. The American QIC, also involving seventeen general practices, led to successful changes in the depression delivery and information system, which were also the most often sustained over time [41]. Organisational structure and leadership support were the most common facilitators, while staff resistance, time constraints, and information technology were the most common barriers.

Our study also relates to several initiatives in the United Kingdom to implement the guidelines of the National Institute for Health and Care Excellence (NICE). The Scottish study, ‘Doing Well’, incorporated the routine use of a depression severity measure with continuous outcome monitoring, a prompt access to guided self-help and a ‘step-up’ to more formal psychological therapy or medical care, if indicated. As a result, daily doses of antidepressants increased less rapidly than in other areas [42]. A British implementation study into stepped care services reported a considerable variation in the design and implementation of the stepped care guideline recommendations [43]. The large scale guideline implementation, Improving Access to Psychological Therapies (IAPT), focused on increasing the availability of evidence-based psychological treatments, both the high intensity therapies (CBT) and the low intensity therapies such as guided self-help, psycho-education groups and behavioural activation [44]. Three year results showed that most patients received guideline-concordant care and that patients had a higher chance of recovery if the treatment sites showed higher step-up rates from low to high intensity treatment in case of insufficient response, as well as if they received an adequate number of sessions [44].

### Implications for research and practice

This study has shown that antidepressant prescribing by GPs can be changed by a multifaceted implementation strategy based on national guidelines and the time and support to implement these in a multidisciplinary context. This message is relevant for clinicians, managers and policy makers, both in Europe and beyond, who are motivated to implement guidelines for depression and to move from an overemphasis on psychopharmacological treatments for depression [45] to stepped depression care, where patients with mild symptoms receive less intensive treatments, such as medication. Policy initiatives aimed at strengthening general practice and reducing unnecessary antidepressant treatment in general practice can use our information, by addressing GPs, psychologists, social workers and specialised mental health nurses to recognise, treat and monitor depression in a stepped care manner, offering guided self-help and brief interventions when possible, and antidepressant medication when necessary [46]. Researchers charged with the task of evaluating such programs are recommended to consider adopting a randomised controlled design, to enable stronger statements about the effect of stepped care approaches and a useful cost effectiveness analysis. Although recruitment of participants to this type of implementation study is challenging, if feasible at all, useful frameworks exist to guide researchers in developing and evaluating these complex interventions [47].

## Conclusions

GPs can learn to change antidepressant prescribing behaviour in the context of an improvement programme. Our study should be considered as one of the first studies focusing on the issue of the over prescribing of antidepressant treatment in general practice. It presents data indicating that GPs can change prescribing behaviour, provided that they have access to alternatives and implementation support. Future implementation studies should expand on this and investigate the stepped care delivery of all depression treatments, recommended in the guidelines. Fortunately, in the Netherlands and beyond, implementation of clinical guidelines followed by process and outcome monitoring for depression are gradually becoming mandatory and better supported by information technology. This is a hopeful message for those trying to improve the care for this patient group.

## Abbreviations

ATC: Anatomical therapeutic chemical; CBT: Cognitive behavioural therapy; CCM: Chronic care model; DSM IV: Diagnostic and statistical manual of mental disorders fourth edition; EMR: Electronic medical record; GDG: Guideline development group; GP: General practitioner; ICPC: International classification of general practice; LINH: Netherlands information network of general practice; METIGG: National ethics committee in mental healthcare; NICE: National institute for health and care excellence; QIC: Quality improvement collaborative.

## Competing interest

The authors declare that they have no competing interests.

## Authors’ contributions

GF carried out the design of the study, the acquisition, analysis and interpretation of data and the draft of the manuscript. JK carried out the statistical analysis of the data and participated in the draft of the manuscript. JH, CvdF and PV have been involved in data interpretation and revising the manuscript for important intellectual content. RG and MW have made substantial contributions to the design of the study, the analysis and interpretation of data and the revision of the manuscript. All authors read and approved the final manuscript.

## Pre-publication history

The pre-publication history for this paper can be accessed here:

http://www.biomedcentral.com/1471-2296/15/35/prepub
